# Aspiration before and after Supraglottoplasty regardless of Technique

**DOI:** 10.1155/2010/912814

**Published:** 2010-11-21

**Authors:** Jeffrey C. Rastatter, James W. Schroeder, Stephen R. Hoff, Lauren D. Holinger

**Affiliations:** ^1^Division of Pediatric Otolaryngology, Children's Memorial Hospital, 2300 Childrens Plaza, Box no. 25, Chicago, IL 60614, USA; ^2^Department of Otolaryngology—Head and Neck Surgery, Feinberg School of Medicine, Northwestern University, Chicago, IL 60611, USA; ^3^Department of Otolaryngology—Head and Neck Surgery, School of Medicine, University of Illinois, Chicago, IL 60612, USA

## Abstract

*Objective*. To determine the incidence of preoperative and postoperative aspiration in infants who undergo supraglottoplasty. To determine the effect of cold steel and CO_2_ laser supraglottoplasty on aspiration in infants with severe laryngomalacia. *Design*. Retrospective study. *Setting*. Tertiary pediatric hospital. * Patients*. Thirty-nine patients who underwent CO_2_ laser-assisted supraglottoplasty (CLS) or cold steel supraglottoplasty (CSS) for severe laryngomalacia. *Main Outcome Measures*. Aspiration and upper-airway obstruction. *Results*. Thirty-nine patients met inclusion criteria (18 males, 21 females). Eighteen patients underwent CSS and 21 patients underwent CLS. 10/39 (25.6%) of the patients had preoperative aspiration, and 2/10 (20%) resolved after supraglottoplasty. New onset aspiration was found in 4/13 (30.8%) in the CSS group and 9/16 (56.3%) in the CLS group. *Conclusions*. There is no significant difference in the rate of postoperative new-onset aspiration or relief of upper-airway obstruction in the CLS or CSS, is temporary and can be managed with thickened diet or temporary tube feedings. The rate of persistent postoperative aspiration was statistically similar regardless of the method of surgery.

## 1. Introduction

Laryngomalacia is the most common congenital laryngeal anomaly and the most common cause of stridor in the infant. Of infants who present with stridor, 60% will have laryngomalacia [1]. Laryngomalacia comprises 50% to 75% of all congenital laryngeal anomalies seen in children [2–4]. In laryngomalacia, a tubular-shaped epiglottis, short aryepiglottic folds, prominent cuneiform cartilages, and redundant arytenoid mucosa cause supraglottic collapse and airway obstruction, particularly with inspiration [5, 6]. 

Laryngomalacia is often diagnosed shortly after birth. The typical course is progression of stridor for 6 months followed by gradual resolution by 12 to 24 months of age [7, 8]. Approximately 10–15% of patients will have severe upper-airway obstruction associated with increased work of breathing, feeding difficulties, and/or failure to thrive [9]. In these severe cases, surgical intervention is indicated. Supraglottoplasty is a well-established method to relieve the airway obstruction, often preventing the need for tracheostomy [10]. Studies have shown that dissection using cold steel instruments or CO_2_ laser successfully relieves airway obstruction in 73% to 95% of cases [11–15]. Success rates have been shown to be lower in children with neurological comorbidities [16].

Feeding disorders can accompany respiratory distress in infants due to disruption of the suck-swallow-breathe sequence. There are reports of coughing and choking in infants with laryngomalacia [16]. Aspiration caused by severe laryngomalacia has recently been reported [17]. There is still some debate as to how supraglottoplasty affects the baseline aspiration caused by laryngomalacia. There is also debate regarding if and to what degree supraglottoplasty may induce aspiration in some infants. Previous studies have shown that 37% of patients have new onset postoperative clinical aspiration with laser-assisted supraglottoplasty [18]. The purpose of this study is to evaluate and compare preoperative and postoperative aspiration rates and feeding difficulties in patients who had supraglottoplasty using either the CO_2_ laser or cold steel dissection. 

## 2. Methods

### 2.1. Patient Selection

The Institutional Review Board for Children's Memorial Hospital approved this retrospective study. Patients who underwent supraglottoplasty for severe laryngomalacia by either author J. W. Schroeder or L. D. Holinger between 2004 and 2008 were identified. Only patients with congenital laryngomalacia were included. Older patients with late onset laryngomalacia or acquired laryngomalacia were excluded. Patients with a history of previous open airway surgery or tracheostomy placement before supraglottoplasty were excluded. Patients with neurological comorbidities were also excluded. 

### 2.2. Data Collection and Analysis

Complete medical records including operative reports, clinical swallow evaluations, and video fluoroscopic swallow study reports were obtained for review. Information collected included age at diagnosis of laryngomalacia, indication for supraglottoplasty, preoperative and postoperative airway obstruction, history of tracheostomy tube placement, and known medical comorbidities. Particular attention was given to identifying neurological comorbidities as this was an exclusion criteria. Neurological comorbidities were defined to include severe developmental delay, cerebral palsy, or a syndrome associated with global neuromuscular dysfunction. Data were also collected regarding preoperative and postoperative feeding difficulties, failure to thrive, and aspiration.

Patients were divided based on technique of supraglottoplasty. The cold steel supraglottoplasty (CSS) group included patients whose surgery was accomplished using only cold steel instruments. The CO_2_ laser supraglottoplasty (CLS) group included patients whose surgery was accomplished using a CO_2_ laser for tissue dissection with or without additional use of cold steel instruments. It should be noted that in our practice during the study period of 2004 to 2008, CLS was the preferred method up to mid-2006, and CSS was the preferred method thereafter. Whether a patient received CLS or CSS, therefore, was based only on the year their surgery occurred. Patients were not selected to receive CLS or CSS based on any individual characteristics related to their medical history, symptom complex, or anatomy.

All patients were evaluated for feeding difficulties and clinical signs of aspiration by the treating otolaryngologist and a speech-language pathologist (SLP). The presence of aspiration was determined by a clinical swallow examination (CSE) with or without a video fluoroscopic swallow study (VFSS). All patients received a postoperative CSE 24 to 48 hours after supraglottoplasty. The CSE is a systemic, complete feeding evaluation and has been shown to be 92% sensitive at diagnosing aspiration of fluids in pediatric patients [19]. Whether or not a patient also got a pre- or postoperative VFSS was decided by the treating SLP as necessary to fully assess aspiration. The SLP had no knowledge regarding the method of supraglottoplasty for any patients. 

### 2.3. Surgical Technique

All patients received a confirmatory awake flexible fiberoptic laryngoscopy. All patients received a rigid bronchoscopy to fully evaluate the tracheobronchial tree and rule out synchronous airway lesions. Supraglottoplasty was performed using suspension microscopic laryngoscopy with a Parsons or Benjamin-Lindholm laryngoscope and visualization with a Zeiss operating microscope (Carl Zeiss, Inc). General anesthesia was achieved using sevofluorane via insufflation technique in a spontaneously breathing child. Supraglottic collapse was treated by incising the aryepiglottic folds, reducing prominent cuneiform cartilages, and reducing excess arytenoid mucosa as previously reported [16, 20]. In the CSS cases, only curved or straight microlaryngeal scissors were used. In the CLS cases, a CO_2_ laser set at 4 watts superpulse mode and a micromanipulator fitted to the Zeiss microscope was used for cutting and tissue dissection. Postoperatively, all patients were treated with IV cefazolin (10 mg/kg q8 hr) and an IV proton pump inhibitor for 24 hours. Patients were given additional 5 to 7 days of antibiotics (Cephalexin or Clindamycin) and at least 1 month of an oral proton pump inhibitor. Patients were observed in the pediatric intensive care unit postoperatively for at least 24 hours. 

### 2.4. Statistical Analysis

The significance of differing rates of postoperative aspiration was determined using logistic regression modeling, adjusting for preoperative aspiration. The postoperative relief of upper airway obstruction was compared using Fisher's exact test. All tests were two sided, with level of significance of *P* < .05. Statistical analysis was conducted using SAS 9.1 (SAS Institute, Cary, NC). Data was compiled, sorted, and analyzed with Microsoft Excel (Microsoft Corporation, Redmond, WA).

## 3. Results

### 3.1. General Patient Characteristics (*n* = 39)

Thirty-nine patients met inclusion criteria (18 males, 21 females). 10/39 (26%) were premature births with a mean gestational age of 31 weeks (range 28–36 wk). Patients were all diagnosed with laryngomalacia by awake flexible fiberoptic laryngoscopy. The mean age at diagnosis of severe laryngomalacia was 12 weeks (range 0.5–60 wk). 18/39 (46.2%) underwent a cold steel supraglottoplasty (CSS group), and 21/39 (53.8%) underwent a CO_2_ laser supraglottoplasty (CLS group). This indication for supraglottoplasty in all patients was severe laryngomalacia causing respiratory distress with or without failure to thrive. Respiratory distress included frequent or severe oxygen desaturations, frequent apnea or bradycardia events, or cyanosis.

#### 3.1.1. Aspiration

In the CSS group, 5/18 (27.8%) had preoperative aspiration, and postoperatively 1/5 (20%) of these patients were no longer aspirated. 13/18 (72.2%) did not have preoperative aspiration, and postoperatively 4/13 (30.8%) developed new aspiration.

**Figure 1:**
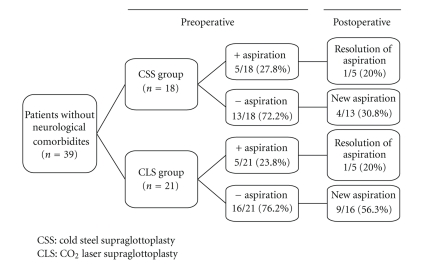
Aspiration in patients without neurological comorbidities.

In the CLS group, 5/21 (23.8%) had preoperative aspiration, and postoperatively 1/5 (20%) of these patients were no longer aspirated. 16/21 (76.2%) did not have preoperative aspiration, and postoperatively 9/16 (56.3%) of these had new aspiration. (Tables 1 and 2, Figure 1).

Preoperatively the diagnosis of aspiration was made by CSE in 27/39 (69.2%) of cases and CSE + VFSS in 12/39 (30.8%) of cases. Postoperatively the diagnosis of aspiration was made by CSE in 8/39 (20.5%) and CSE + VFSS in 31/39 (79.5%) of cases. No statistically significant difference was present between the CSS group and the CLS group with respect to new onset aspiration (*P* = .18, Table 1).

#### 3.1.2. Resolution of Upper-Airway Obstruction

In the CSS group, 18/18 (100%) had improvement of upper-airway obstruction, and 0/18 (0%) required a tracheotomy tube for persistent severe upper-airway obstruction. In the CLS group, 18/21 (85.7%) had improvement of upper-airway obstruction, and 3/21 (14.3%) required a tracheotomy tube for persistent severe upper-airway obstruction. Overall for all patients in this population, 36/39 (92.3%) had improvement of upper-airway obstruction, and 3/39 (7.7%) required a tracheotomy tube (Table 3). No statistically significant difference was present between the CSS group and the CLS group with respect to resolution of upper-airway obstruction (*P* = .61).

## 4. Discussion

Laryngomalacia remains a commonly diagnosed condition in the pediatric population. Approximately 10% to 15% of cases of laryngomalacia will have severe upper-airway obstruction. These severe cases can benefit from surgical intervention [9]. Supraglottoplasty has been shown to be highly successful at relieving upper-airway obstruction and dysphagia in these severe cases.

Advancements in surgical techniques for supraglottoplasty have been made. Relatively aggressive techniques such as epiglottectomy [21] have been replaced by the more conservative and less morbid endoscopic techniques of epiglottoplasty and supraglottoplasty [8, 10, 22]. Over the last 20 years, endoscopic techniques have become the standard. The CO_2_ laser has been used to perform supraglottoplasty since 1985, and it is still commonly used for this purpose today [10].

Supraglottoplasty will resolve the upper-airway obstruction caused by laryngomalacia in 73% to 95% of cases [11–15]. Patients with significant neurological comorbidities tend to do worse after supraglottoplasty [16]. In this current study, patients with neurological comorbidities were excluded. Excluding these patients was necessary to maintain a relatively uniform population to study the difference in outcome between CSS and CLS. If included, patients with neurological comorbidities would be expected to have higher rates of postoperative aspiration regardless of the method of surgery. This would confound the main outcome measure of postoperative aspiration as we cannot assume uniform distribution of neurological comorbidities between the groups in a retrospective study.

We concluded that CSS and CLS did not show a significant difference in the rate of relief of upper-airway obstruction due to severe laryngomalacia (100% and 85.7%; *P* = .61). The overall rate of 92.3% of improved upper airway obstruction is consistent with other series [11–15] (Table 3).

Operating on the supraglottis carries a risk of postoperative aspiration and feeding difficulties [18]. Minor disruptions in upper aerodigestive anatomic and neuromuscular control in an infant can contribute to respiratory and feeding issues that potentially result in aspiration [23]. Airway obstruction from prolapsing supraglottic tissue may alter the suck-swallow-breathe sequence, leading to aspiration. Furthermore, alterations in normal laryngeal sensation, recently revealed in infants with laryngomalacia, may also contribute to aspiration [16]. In our study, 10/39 (25.6%) of cases had severe laryngomalacia and aspiration prior to surgical intervention. This is supported by recent clinical evidence of preoperative aspiration caused by laryngomalacia. Richter et al. report that 72% of infants with laryngomalacia that required surgical management had evidence of aspiration on Functional Endoscopic Evaluation of Swallowing (FEES) before supraglottoplasty [17]. Laryngeal penetration occurred slightly more frequently at 88% [17]. 

In some cases, supraglottoplasty will improve dysphagia and lead to resolution of aspiration. In our study, 1/5 (20%) of patients with preoperative aspiration improved after CSS, and 1/5 (20%) with preoperative aspiration improved after CLS. No statistically significant difference was present between these groups. Using FEES examinations, Richter et al. determine that 86.1% of cases of preoperative aspiration resolved after supraglottoplasty when cold knife surgical technique was used [17]. They postulate that improvement in airway patency through supraglottoplasty decreased the work of breathing and enhanced the coordination of the suck-swallow-breathe sequence. Therefore, the risk of aspiration was reduced [17]. It is unclear why their rate of resolution differs with our findings. It may be due to the method used to evaluate aspiration (FEES versus VFSS/CSE) or it may be that our surgical method was less aggressive. 

We also reviewed the rate of new-onset aspiration after supraglottoplasty. Of the total study patients, 29/39 (74.4%) did not have preoperative aspiration. 13/29 (44.8%) developed aspiration after supraglottoplasty. This is consistent with previous reports [18]. Patients in the CLS group had a higher rate of postoperative new-onset aspiration than the CSS group (9/16 [56.3%] versus 4/13 [30.8%]); however, the difference was not significant (*P* = .18). 

We routinely have patients evaluated by an SLP at 24 to 48 hours after supraglottoplasty. Cases of relatively *brief *post operative new onset aspiration are included with the smaller number of *prolonged* new-onset aspirators in regards to the overall calculated rate in this study. Due to the retrospective nature of this current study, precisely calculating the length of postoperative aspiration was not possible do to the number of patients lost to followup. In general, most postoperative aspiration in patients resolved in less than 3 months and was managed by conservative measures such as thickened diet and on occasion brief tube feeding. Three patients had a prolonged tube feeding requirement of more than one year. All three eventually were transitioned to an oral diet. More in-depth examination of the degree and duration of postoperative new-onset aspiration is a necessary topic for future study.

Severe laryngomalacia to the degree that supraglottoplasty is indicated is relatively rare, and studies often have low numbers of patients. It is possible that type 2 statistical error is present in this study, that would suggest no difference between the groups when indeed there is. Larger prospective, multi-institutional studies are needed to address this issue. Based on results from this study, however, we cannot recommend one method of surgery, CLS or CSS, as being superior in regard to postoperative aspiration risk. 

Supraglottoplasty remains an essential procedure for managing severe laryngomalacia. It can provide relief of upper-airway obstruction in 90% of cases. We report the presence of preoperative aspiration with a less dramatic resolution of aspiration after supraglottoplasty than previously reported. We also report the presence of new-onset clinical aspiration after supraglottoplasty regardless of the surgical method used. At our institution, regardless of method of supraglottoplasty, a clinical swallow examination is routinely performed preoperatively and at the first postoperative feeding. A video fluoroscopic swallow study is ordered as clinically indicated. Proper feeding regimens are then adjusted and followed closely by otolaryngology and speech pathology.

## 5. Conclusions

A significant number of patients with severe laryngomalacia have aspiration prior to surgical intervention. In some patients, aspiration will resolve after supraglottoplasty. There is no statistically significant difference in the rate of new-onset postoperative aspiration or relief of upper-airway obstruction in the CLS group compared to the CSS group. Most postoperative aspiration, regardless of using CLS or CSS, is temporary and can be managed with thickened diet or temporary tube feedings. The rate of persistent aspiration after supraglottoplasty was similar regardless of the method of surgery. Based on this study, we cannot recommend CLS or CSS as a preferred method for supraglottoplasty.

## Figures and Tables

**Table 1:** New Onset Aspiration.

		Patients without preoperative aspiration*	New onset aspiration**
Without neurological comorbidities	CSS (*n* = 18)	*n* = 13	4/13 (30.8%)
	CLS (*n* = 21)	*n* = 16	9/16 (56.3%)
			*P* = .18

CSS: cold steel supraglottoplasty; CLS: CO_2_ laser supraglottoplasty.

*Number of patients in a group without evidence of preoperative aspiration.

**Postoperative aspiration in patients who were not aspirating preoperatively.

**Table 2:** Resolution of Aspiration.

		Patients with preoperative aspiration*	Resolution of aspiration**
Without neurological comorbidities	CSS (*n* = 18)	*n* = 5	1/5 (20%)
	CLS (*n* = 21)	*n* = 5	1/5 (20%)
			*P* = .99

CSS: cold steel supraglottoplasty; CLS: CO_2_ laser supraglottoplasty.

*Number of patients in a group with evidence of preoperative aspiration.

**No postoperative aspiration in patients who were aspirating preoperatively.

**Table 3:** Postoperative Relief of Upper Airway Obstruction: CSS versus CLS.

		Improved UAO	Persistent severe UAO requiring tracheostomy
Without neurological comorbidities	CSS (*n* = 18)	100%	0%
	CLS (*n* = 21)	85.7%	14.3%
		*P* = .61	*P* = .61

CSS: cold steel supraglottoplasty; CLS:  CO_2_ laser supraglottoplasty; UAO: upper airway obstruction.
